# Traditional Chinese medicine for mild-to-moderate ulcerative colitis: Protocol for a network meta-analysis of randomized controlled trials

**DOI:** 10.1097/MD.0000000000016881

**Published:** 2019-08-16

**Authors:** Zhaofeng Shen, Qing Zhou, Yingjun Ni, Weiming He, Hong Shen, Lei Zhu

**Affiliations:** a Department of Science and Technology, Jiangsu Province Hospital of Chinese Medicine; b School of Public Health, Nanjing Medical University; c Department of Gastroenterology, Jiangsu Province Hospital of Chinese Medicine; d Department of Pharmacy, Jiangsu Province Hospital of Chinese Medicine, Affiliated Hospital of Nanjing University of Chinese Medicine, Nanjing, Jiangsu, China.

**Keywords:** network meta-analysis, protocol, Traditional Chinese medicine, ulcerative colitis

## Abstract

**Background:**

Ulcerative colitis (UC) is a universal chronic nonspecific intestinal inflammatory disease of unknown etiology. Although 5-aminosalicylic acid (5-ASA) is used as a first-line treatment for mild-to-moderate UC, some patients do not react well to it. Traditional Chinese medicine (TCM) plays a complementary role in the management of UC. A large number of randomized controlled trials (RCTs) have shown that TCM has a significant effect in the treatment of mild-to-moderate UC. However, due to the diversity of TCM treatments, its relative effectiveness and safety remains unclear. Therefore, we aim to compare the effectiveness and safety of TCM for mild-to-moderate UC by implementing a Bayesian network meta-analysis (NMA) and provide a reference for clinical treatment.

**Methods:**

According to the Cochrane Handbook, PubMed, MEDLINE, Embase, Web of Science, the Cochrane Library, CINAHL, China National Knowledge Infrastructure (CHKD-CNKI), Chinese Biomedical Literature database (CBM), and WANFANG database will be searched. Related randomized controlled trials (RCTs) that compared one TCM intervention with another or with 5-ASA (placebo) for mild-to-moderate UC from inceptions to February 2019 will be included. Two authors will screen the literature and extract data independently based on predesigned rules, and evaluate the risk of bias of included studies using the Cochrane Risk of Bias Tool. Both classical pair-wise meta-analysis and Bayesian NMA will be conducted using R-3.4.4 and WinBUGS-1.4.3 software. The ranking probabilities for all interventions will be estimated and the hierarchy of each intervention will be summarized as surface under the cumulative ranking curve. The consistency within network will be evaluated with Cochrane Q statistic and net-heat plot. The quality of evidence will be assessed by the Grading of Recommendations, Assessment, Development and Evaluation (GRADE) approach.

**Results:**

The study results will be disseminated through a peer-reviewed journal publication or conference presentation.

**Conclusions:**

The findings will provide a systematic evidence-based medical evidence of TCM interventions in the treatment of UC and help clinical practitioners, UC patients, and policy-makers make more informed choices in the decision-making.

**Ethics and dissemination:**

Ethical approval and informed consent are not required since this is a protocol for a network meta-analysis based on published studies. The findings will be disseminated through a peer-reviewed journal publication or conference presentation.

**Registration:**

PROSPERO CRD42019133962.

## 1 Introduction

Ulcerative colitis (UC), a subtype of inflammatory bowel disease (IBD), is a chronic idiopathic intestinal inflammatory disease caused by multiple factors. This condition has a tendency of recurrence and a long course of the disease throughout life.^[1]^ Its clinical manifestations are complex and varied, such as abdominal pain, diarrhea, and bloody stool, etc. It is reported that the incidence rates of UC in Europe and North America were as high as 24.3/10 million and 19.2/10 million respectively, and the prevalence rates reached 505/10 million and 249/10 million respectively.^[2]^ As a consequence, they are at a plateau.^[3]^ However, the incidence rates of UC in newly industrialized countries are increasing year by year. According to an epidemiological survey, there were about 350,000 new cases of IBD in China from 2005 to 2014. By 2025, the number of IBD patients in China will reach 1.5 million.^[4]^ As the pathogenesis mechanisms of UC are not completely understood, UC has emerged as a public health challenge worldwide. Because of the lack of curative treatment, the main goal of medical therapy in UC is to control the acute onset of the disease, induce and maintain remission, heal the mucosa, and reduce recurrence.^[5]^ Currently, the medical treatment for UC relies mainly on anti-inflammatory drugs, including 5-aminosalycilate compounds, corticosteroids, and immunosuppressants.^[6]^ For patients with mild-to-moderate UC, 5-ASA (oral or topical administration) is recommended as a first-line therapy. Patients who can not achieve remission should be treated with corticosteroids.^[7]^ But those drugs often result in side effects, which may reduce health-related quality of life,^[8]^ particularly during long-term treatment.^[9]^

Under the circumstance, traditional Chinese medicine (TCM), an important part of complementary and alternative medicine (CAM), plays an irreplaceable role in the treatment of UC. The use of TCM in patients with UC has increased in popularity over the past several decades because of the unique advantages of efficacy, convenience, safety, and low cost. Recently, an increasing amount of evidenced-based medicine (EBM) evidences have shown that TCM therapies including Chinese herbal medicine, Chinese patent medicine, acupuncture, moxibustion, massage, and acupoint application, have potentially positive effects on UC.^[10,11,12,13,14]^ Although previous systematic reviews had shown sound effects of TCM for treating UC patients, the quality of the studies has become a common concern. To make matters worse, no comparative effectiveness investigation was done before, which may exert some influence on clinical decision-making. As a result, there is no consensus on which TCM therapy should be chosen as a first option properly. Therefore, further researches are needed in order to clarify the relative efficacy and safety of TCM therapies for UC. In this study, we will conduct a systematic review incorporating Bayesian NMA to systematically compare the efficacy and safety of different TCM interventions, paving the way for future avenues to resolve ulcerative colitis.

## 2 Methods

### 2.1 Design and registration

The protocol follows the Cochrane Handbook for Systematic Reviews of Interventions and the Preferred Reporting Items for Systematic Reviews and Meta-Analysis Protocol (PRISMA-P)^[15]^ statement guidelines. The reporting of the following NMA will obey the PRISMA extension statement for reporting of systematic reviews incorporating network meta-analysis of healthcare interventions.^[16]^ This protocol has been registered on PROSPERO CRD 42019133962 (https://www.crd.york.ac.uk/PROSPERO/). No further ethical approval is required since all eligible studies were approved by local institutional review boards and ethical committees.

### 2.2 Information sources and search strategy

We will systematically search PubMed, Web of Science, MEDLINE, CINAHL, the Cochrane Library, Embase, China National Knowledge Infrastructure (CHKD-CNKI), Chinese Biomedical Literature database (CBM), and WANFANG database for related randomized controlled trials (RCTs) that compared one TCM intervention with another or with 5-ASA (placebo) in the treatment of mild-to-moderate UC. The temporal interval is limited from the time that the databases created to February 2019.

The searches were restricted to papers that were published in English or Chinese. The search strategy will be conducted independently by 2 authors (SZF and ZQ) who are experienced in the information retrieval and combine free text words and medical subject headings regarding “Chinese medicine,” “traditional Chinese medicine,” “Chinese herbal,” “Ulcerative colitis,” “ UC,” “Inflammatory bowel disease,” “IBD,” and “randomized controlled trials.” MeSH and subheadings were combined with “AND” or “OR.” Furthermore, we will also retrieve the WHO International Clinical Trials Registry Platform and ClinicalTrials.gov to identify ongoing trial registers. We will manually search related systematic reviews/meta-analyses and bibliographies of included studies to identify additional potential studies. The preliminary search strategy for PubMed is summarized in Table 1, which will be adapted according to syntax-related requirements of other electronic databases.

**Table 1:**
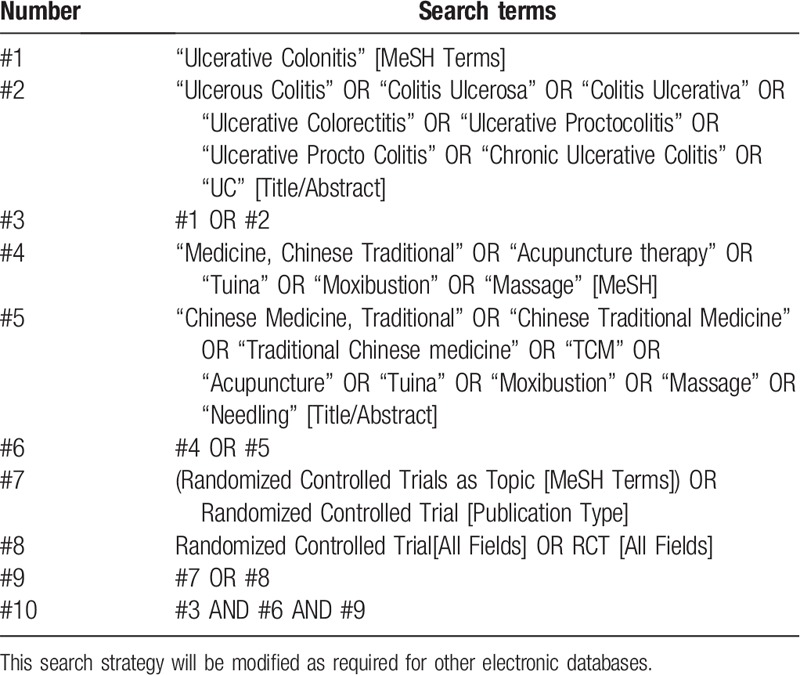
Search strategy used in PubMed database.

### 2.3 Eligibility criteria

The PICOS (Participant-Intervention-Comparator-Outcome-Study design) framework will be applied in the study design.

#### 2.3.1 Type of study

We will include all randomized controlled trials (RCTs) of TCM for UC regardless of blinding or allocation concealment. The language of the literature will be limited to Chinese or English. Studies with non-RCTs (case reports, case-control studies, Cohort studies, and reviews), quasi-RCTs, and animal experiments will be excluded,^[17]^ while cluster RCTs will be included when the clustering effect can be taken account of. Although increasing numbers of clinical trials have been reported from mainland China,^[18]^ there is a concern about their quality.^[19,20]^ Therefore, we will include Chinese trials as long as they were approved by local institutional review boards and registered in an international database.

#### 2.3.2 Participants

Patients with a confirmed clinical, endoscopic, and histological diagnosis of mild-to-moderate ulcerative colitis using any recognized diagnostic criteria will be included. There are no limitation in age, sex, nation, ethnicity, and disease stage. However, studies that enrolled participants with chronic bacterial dysentery, chronic amoebic bowel disease, schistosomiasis, and so on, will be excluded.

#### 2.3.3 Interventions

We will include studies in which the TCM interventions have been applied, including Chinese herbal medicine, Chinese patent medicine, acupuncture, moxibustion, massage, and acupoint application, etc. Eligible treatments can be used as monotherapy and combined treatments, or in combination with Western medicine (5-aminosalicylic acid). The intervention should be performed consecutively for at least for 4 weeks. Any studies including other Western medicines will be excluded.

#### 2.3.4 Comparators

The control group is treated with other TCM interventions, 5-aminosalicylic acid (5-ASA, drugs recommended in international authorized clinical guidelines), or placebo.

#### 2.3.5 Outcomes

The primary outcomes are Clinical efficiency and clinical remission rates from baseline to the last available follow-up, measured using the Mayo score. The secondary outcomes will include endoscopic response rate, mucosal healing rate, quality of life assessed using the IBDQ (Inflammatory Bowel Disease Questionnaire), and the safety of the intervention, including any associated adverse effects.

### 2.4 Study selection and data extraction

**Figure 1:**
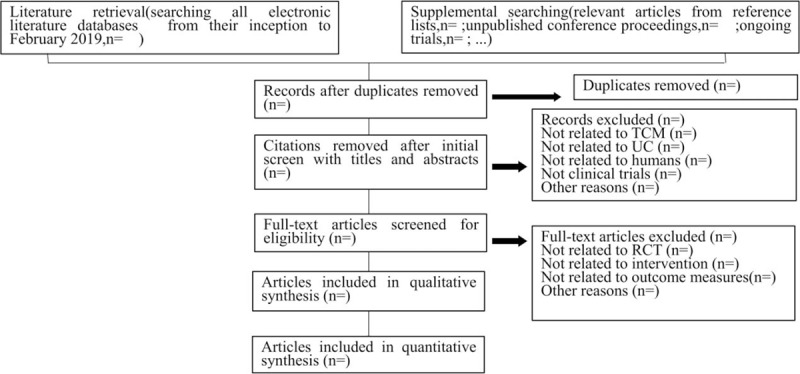
Study flow chart. This flow chart is based on PRISMA framework, which shows the whole process of literature retrieving, screening, inclusion and exclusion. PRISMA = Preferred Reporting Items for Systematic Reviews and Meta-Analyses.

Two researchers will independently conduct the literature retrieval, literature screening, data extraction and quality evaluation procedures. In case of disagreements, they will consult other researchers and negotiate using the original data. The 2 researchers will identify relevant literature by reading the titles, abstracts, and full-texts of the studies retrieved during the searches with reference to the eligibility criteria mentioned above. The process of study selection will be summarized in the PRISMA flowchart^[21]^ in Fig. 1. The search results will be managed by NoteExpress 2.0 (http://www.inoteexpress.com). The following information will then be extracted from the studies selected for inclusion using a preestablished literature extraction table: author, article title, year of publication, contact information, country, sample size, participants, diagnosis criteria, baseline characteristics, study design, randomization method, blinding, experimental intervention, control intervention, duration, treatment frequency, outcomes, adverse events, etc. The original authors of any articles which are found to have missing information will be contacted as much as possible in an attempt to obtain the data, or to perform data conversion. If the data does not prove to be available, the study in question will be discarded.

### 2.5 Risk of bias assessment

Two review authors (SZF and ZQ) will independently assess the risk of bias (methodological quality) of each included study in duplicate using the bias risk assessment tool^[22]^ recommended in the Cochrane Handbook for Systematic Reviews of Interventions 5.1.0, which comprises 7 items: random sequence generation, allocation concealment, blinding of participants and personnel, blinding of outcome assessment, incomplete outcome data, selective reporting, and other bias. The results for each domain will be divided into 3 levels: low risk of bias, high risk of bias, and unclear risk of bias,^[23]^ and studies will be assessed and given an overall rating. Any inconsistencies between the reviewers will be resolved through discussion or, if necessary, a third reviewer (ZL) will be consulted.

### 2.6 Grade of evidence

In addition, the quality of the evidence will be evaluated by the Grading of Recommendations Assessment, Development, and Evaluation (GRADE) approach,^[24]^ which will be classified into four levels (high quality, moderate quality, low quality, and very low quality) according to 5 domains including risk of bias, inconsistency, indirectness, imprecision, and publication bias.

### 2.7 Data synthesis and statistical analysis

We will perform a conventional pairwise meta-analysis of the direct evidence using the random-effects model (Hartung-Knapp-Sidik-Jonkstra method)^[25]^ with R 3.4.4 software (https://cran.r-project.org/src/base/R-3/) to estimate the effect size and 95% CI. Then, a network meta-analysis (NMA) within a Bayesian framework will be undertaken using WinBUGS 1.4.3 (MRC Biostatistics Unit, Cambridge, UK) software. We will adopt random-effects and consistency models, as they are considered to be the most conservative approach to dealing with between-study heterogeneity.^[26]^ A 2-stage approach to the data synthesis will be used. For dichotomous outcomes, data will be analyzed by calculating Mantel-Haenszel odds ratios (OR, LOR) together with 95% confidence intervals (CIs), where appropriate. For continuous outcomes, standardized mean difference (SMD) and 95% CIs will be used to summarize the data for each group.

R 3.4.4 and WinBUGS 1.4.3 software will be used to perform the NMA to compare direct and indirect evidence. Through the construction and verification of the model by WinBUGS, the random effects Markov Chain Monte Carlo (MCMC) will be used to simulate the data, and 3 chains, and different iterations (number of annealing times) will be set. To generate posterior distributions of model parameters, 150,000 iterations of MCMC after 50,000 tuning iterations in 3 chains will be run.^[27]^ Through the posterior distribution kernel density estimation, Gibbs sampling dynamic trajectory, iterative history map and error information criterion (DIC), the convergence and degree of fit of the model will be judged. We will examine the consistency of NMA by using the node-splitting analysis method. For a closed loop of three treatments, the inconsistency between direct and indirect evidence will be directly assessed. For the closed loop formed by the 4 studies, it can be divided into 2 closed triangular loops to test the inconsistency. Inconsistency factors value (IF) will be used to calculate the absolute difference. Clinical and methodological heterogeneity will be appraised by checking the characteristics and design of the included studies. The degree of statistical heterogeneity will be quantified by the *I* ^2^ statistic. To examine the potential of small-study effects in the network, comparison-adjusted funnel plots will be produced.^[28]^ Besides, if possible, sensitivity analysis will be explored to assess the robustness of outcomes. We will use meta-regression and sensitivity analysis to evaluate the impact of covariates and the source of heterogeneity.

Furthermore, geometry of the network will be drawn to present the structure of interventions across studies to ensure the feasibility of the NMA. The probability of each intervention being the best for each outcome will be calculated and reported in the form of rankograms.^[28]^ The hierarchy of interventions will be ranked by importing the above model into R software and calculating the surface under the cumulative ranking curve (SUCRA).

## 3 Discussion

In recent years, publications of clinical RCTs that compared TCM intervention with 5-ASA for patients with UC have been increasing continuously. Many studies have shown that Chinese medicine is not inferior to western medicine (5-ASA) in the treatment of mild-to-moderate UC. Nevertheless, there are few studies which directly compare among different TCM therapies. TCM has a profound theoretical foundation and rich clinical experience in the treatment of mild-to-moderate UC. Although the specific mechanism of TCM treatment of UC is not completely clear, it is believed that TCM therapy can stimulate the body's righteousness and regulate the balance of qi and blood, besides, yin and yang. At present, there is no unified normalized standard and therapeutic principle of TCM for UC. Given that systematic reviews with good quality may provide best evidence for clinical practice, we would like to perform a NMA to figure out which TCM intervention has the relatively optimal effect and safety.

This protocol has been registered with international prospective register of systematic reviews (PROSPERO CRD 42019133962). And it will follow the guidelines of Cochrane Handbook for Systematic Reviews of Interventions and the PRISMA-P statement. Moreover, the quality of evidence will be appraised by the GRADE approach. However, there are certain limitations of our NMA, including publication bias, clinical heterogeneity, and selection bias.

In summary, we hope this study can provide a possible ranking for TCM treatment of mild-to-moderate UC and give some new insight into the treatment of UC to some extent.

## Author contributions

**Conceptualization:** Zhaofeng Shen, Weiming He, Hong Shen, Lei Zhu.

**Data curation:** Zhaofeng Shen, Yingjun Ni.

**Formal analysis:** Zhaofeng Shen, Qing Zhou, Lei Zhu.

**Funding acquisition:** Zhaofeng Shen, Hong Shen, Lei Zhu.

**Investigation:** Zhaofeng Shen, Qing Zhou, Yingjun Ni.

**Methodology:** Zhaofeng Shen.

**Project administration:** Zhaofeng Shen, Qing Zhou, Yingjun Ni, Lei Zhu.

**Resources:** Zhaofeng Shen, Hong Shen, Lei Zhu.

**Software:** Zhaofeng Shen, Yingjun Ni.

**Supervision:** Weiming He, Hong Shen.

**Validation:** Qing Zhou, Yingjun Ni.

**Visualization:** Zhaofeng Shen.

**Writing – original draft:** Zhaofeng Shen, Qing Zhou, Hong Shen, Lei Zhu.

**Writing – review & editing:** Zhaofeng Shen, Qing Zhou.
